# Strengths and Limitations of Using the Polypill in Cardiovascular Prevention

**DOI:** 10.1007/s11886-017-0853-y

**Published:** 2017-04-19

**Authors:** Ambuj Roy, Nitish Naik, K. Srinath Reddy

**Affiliations:** 1 President, Public Health Foundation of India, New Delhi, India; 2 Public Health Foundation of India, Delhi National Capital Region, Plot No. 47, Sector 44, Institutional Area, Gurugram, 122002 India

**Keywords:** Polypill, Fixed dose combination, Cardiovascular diseases, Adherence, Secondary prevention, Primary prevention

## Abstract

**Purpose of Review:**

Polypill and its role in cardiovascular disease (CVD) prevention has been extensively discussed and debated since the inception of the concept in 2003. This article reviews the subsequent accumulated research in this area.

**Recent Findings:**

Several short and intermediate to long-term studies with different brands of polypills have analysed the impact of polypill in phase II and III trials. The strengths of polypill that have emerged include better adherence, equivalent or better risk factor control and quality of life among polypill users as compared to usual care. The lurking limitations include difficulty with dose adjustment to targets, fear of mass medicalisation and low acceptability among physicians.

**Summary:**

The current literature supports polypill use in reducing blood pressure and cholesterol levels for CVD prevention with improvement in adherence to medication. However, the long-term outcome of polypill on CVD events and mortality are unavailable and are currently being studied in clinical trials.

## Introduction

The concept of polypill or a fixed drug combination (FDC) was proposed, to reduce cardiovascular burden, rather dramatically in a much cited paper by Wald and Law in the year 2003 [1]. They proposed that this polypill comprising of six drugs (aspirin, statin, beta-blocker, angiotensin converting enzyme (ACE) inhibitor, diuretic and folic acid) could reduce burden of cardiovascular disease (CVD) by 80% when taken by all above the age of 55 years and those with CVD. The concept was based on research accumulated over years on the benefit of blood pressure lowering, cholesterol lowering and antiplatelet drugs in reducing CVD morbidity and mortality. Over the last decade or so the story of the polypill has progressed with both successes and disappointments [2•]. This review discusses the potential role of polypharmacy, the current state of research, strengths and limitations and the future directions that we foresee for the polypill.

## Importance of Polypharmacy in CVD Prevention

CVD is the leading cause of mortality worldwide [3]. This is true of both the developed world and most of the developing countries. While CVD burden is declining in the developed countries, it is on the rise in developing countries with 80% of the burden projected to be in the lower and middle income countries (LMIC) by 2020 [4].

All current guidelines recommend aspirin and high dose statins for patients with established cardiovascular disease [5, 6]. In addition most patients with CVD are advised beta blockers and ACE inhibitors as class I or IIA indication unless specifically contraindicated [5]. Modelling and nested case control studies have shown that this combination pharmacotherapy leads to a 75% reduction in mortality in patients with established CAD [7].

In patients without CVD, the strength of evidence for polypharmacy is not well established. While the benefits of management of individual risks like hypertension, dyslipidemia are well documented general use of aspirin, and statins remains controversial. Aspirin trials for primary prevention reduce non-fatal MI but have little impact on mortality, while consistently causing increase in risk of bleeding [8]. Similarly, the utility/efficacy of statin for primary prevention is not well established. Two large primary prevention trials did show benefit of use of rosuvastatin in reducing cardiovascular outcomes in individuals without CVD [9, 10]. However, they used a biomarker or clinical risk identifier to include at risk individuals to administer statin. Similarly, appropriate risk stratification strategy to identify high risk individuals for primary prevention with polypill needs to be tested before adopting it. This could be based on clinical scores [10] or novel investigations like calcium score, which has been claimed by authors in a modelling paper, to substantially bring down the number to treat (NNT) [11] as compared to basing it only on age as proposed by Wald and Law.

## Summary of Current Research

The last decade has witnessed development of multiple polypills, with varying constituents, which have been tested in different trials. The polypills considered here for CVD prevention are those with at least one anti-hypertensive in addition to aspirin and statin. A list of these polypills studied or marketed is documented in Table 1.

**Table 1:** List of currently available polypills for research and clinical use

Brand name	Constituents	Manufacturer
Red Heart Pill™ 1	Aspirin (75 mg), atenolol (50 mg), lisinopril (10 mg), simvastatin (40 mg)	Dr. Reddy’s Laboratories,India
Red Heart Pill™ 2	Aspirin (75 mg), hydrochlorothiazide (12.5 mg), Lisinopril (10 mg), simvastatin (40 mg)	Dr. Reddy’s Laboratories, India
Trinomia^®^/Sincronium^®a^	Aspirin (100 mg), ramipril (2.5, 5 or 10 mg), atorvastatin (20 mg)	Ferrer Internacional, Spain
Trinomia^®^	Aspirin (100 mg), ramipril (2.5,5 or 10 mg), simvastatin (40 mg)	Ferrer Internacional, Spain
Polycap^®^	Atenolol (50 mg), hydrochlorothiazide (12.5 mg), ramipril (5 mg), simvastatin (20 mg), optional aspirin (100 mg)	Cadila Pharmaceuticals Ltd., India
Starpill^®^	Aspirin (75 mg), losartan potassium (50 mg), atenolol (50 mg), atorvastatin (10 mg)	Cipla, India
Polypill^b^	Amlodipine (2.5 mg), losartan (25 mg), hydrochlorothiazide (12.5 mg), simvastatin (40 mg)	Cipla, India
PolyIran	Aspirin (81 mg), enalapril (5 mg); or valsartan (40 mg), hydrochlorothiazide (12.5 mg), atorvastatin (20 mg)	Alborz Darou Pharmaceutical Company, Iran
Ramitorva^®^	Aspirin (75 mg), ramipril (5 mg), atorvastatin (10 mg)	Zydus Cadila, India

^a^In Germany, named Sincronium^®^ and manufactured by Hexal

^b^No brand name given

Adapted from [2•]

These polypills have been studied in numerous research trials in comparison to placebo or usual care. These clinical trials have measured adherence rates, adverse events and risk factor control. None of these trials have enough power to test the impact of polypill on clinical outcomes. These are summarised in Table 2, and the different aspects of the trial are discussed below.

**Table 2:** Summary of current studies with polypill

Study title	Countries	Polypill brand	Clinical trial design	Patients	Study groups	Study Duration	Study highlight
IMPACT [12] (2014)	New Zealand	Red Heart Pill™ version 1 or 2	Randomised, open label	513 patients at high risk (>15%) of CVD	Polypill vs. usual care	Minimum12 months	Adherence to all four recommended drugs was greater among polypill participants (81% vs. 46%; *p* < 0.001)
PILL [13] (2011)	Australia, Brazil, India, The Netherlands, New Zealand, UK, USA	Red Heart Pill™ 2	Randomised, double-blind, placebo-controlled	378 Patients with 7.5% estimated 5-year CVD risk	Polypill vs. placebo	12 weeks	Polypill treatment reduced SBP by 9.9 mmHg and LDL-cholesterol by 0.8 mmol/L. The lowering of predicted cardiovascular risk was moderately lower than previous estimates by modelling
Wald et al. [14] (2012)	UK	Cipla polypill^13^	Randomised, placebo-controlled double-blind	86 patients aged 50 years or over with no history of CVD	Polypill vs. placebo	12 weeks	Polypill resulted in a 17.9 mmHg (95% CI 15.7 to 20.1) lower mean systolic BP and 54.1 mg/dl (95% CI 1.2 to 1.6) lower mean LDL cholesterol
TIPS-1 [15] (2009)	India	Polycap™	Randomised, double-blind	2053 individuals without CVD with one risk factor	Polypill vs. eight other usual care groups	16 weeks	Polycap was as effective in reducing SBP/DBP and having aspirin action as individual drugs. It also reduced LDL cholesterol but marginally less than simvastatin alone.
TIPS-2 [16] (2012)	India	Polycap™	Randomised, double blind, 2X2, factorial, controlled	518 patients with previous CVD or diabetes mellitus	Single-dose polypill plus placebo or two polypill capsules plus k+	8 weeks	Two polypills reduced BP further by 2.8/1.7 mmHg, LDL-C by 6.6 mg/dl with no increase in drug discontinuation rates
TEMPUS [17] (2015)	The Netherlands	Red Heart Pill™ Version 2	Randomised, open, blinded end-point, three-period, crossover	78 patients with established CVD	Morning polypill vs. evening polypill vs. usual care	3–6 weeks per period	The use of a polypill in the evening was more effective in lowering LDL-cholesterol but not BP as compared to morning polypill. Polypill had higher adherence rates and high rate of preference
UMPIRE [18] (2013)	India and Europe (UK, Ireland, and The Netherlands)	Red Heart Pill™ Version 1 or 2	Randomised, open-label, blinded end point	2004 patients with CVD or at high risk (>15%) of CVD	Polypill vs. usual care	24 month (minimum 12 month)	Polypill resulted in 33% improved adherence at 15 months and small but significant improvements in SBP and LDL-C.
Kanyini-GAP [19] (2015)	Australia	Red Heart Pill™ Version 1 or 2	Randomised, open-label	623 patients with or at high risk (>15%) of CVD	Polypill vs. usual care	34 months (minimum 12 months)	After a median of 18 months, the polypill-based strategy was associated with greater use of combination treatment
SPACE [20••] (2016)	As in IMPACT, UMPIRE and Kanyini-GAP	Red Heart Pill™ Version 1 or 2	Meta-analysis	3140 patients from IMPACT, UMPIRE and Kanyini-GAP studies	Polypill vs. usual care	12 months	Polypill arm had higher adherence to combination therapy (80% vs. 50%, RR 1.58; < 0.001), lower SBP (2.5 mmHg; *p* = 0.02) and lower LDL-cholesterol (3.9 mg/dl; *p* = 0.04). The benefit was most among those who were under-treated at baseline.
FOCUS Phase II [21] (2014)	Italy, Spain, Argentina, Paraguay	Trinomia™	Randomised, open-label, active-controlled, piggy back, parallel	695 post-MI patients	Polypill vs. usual care	9 months	Polypill group had better adherence after 9 months of follow-up: 50.8% versus 41% (*p* = 0.019)

*FOCUS* Fixed-Dose Combination Drug for Secondary Cardiovascular Prevention, *IMPACT* IMProving Adherence using Combination Therapy, *PILL* Programme to Improve Life and Longevity, *SPACE* Single Pill to Avert Cardiovascular Events, *TIPS* Trauma Informed Personalised Scripts, *UMPIRE* Use of a Multidrug Pill In Reducing Cardiovascular Events, *SBP* systolic blood pressure, *DBP* diastolic blood pressure, *LDL* low density lipoprotein

Adapted from [2•]

## Strengths

### Adherence

**Fig. 1:**
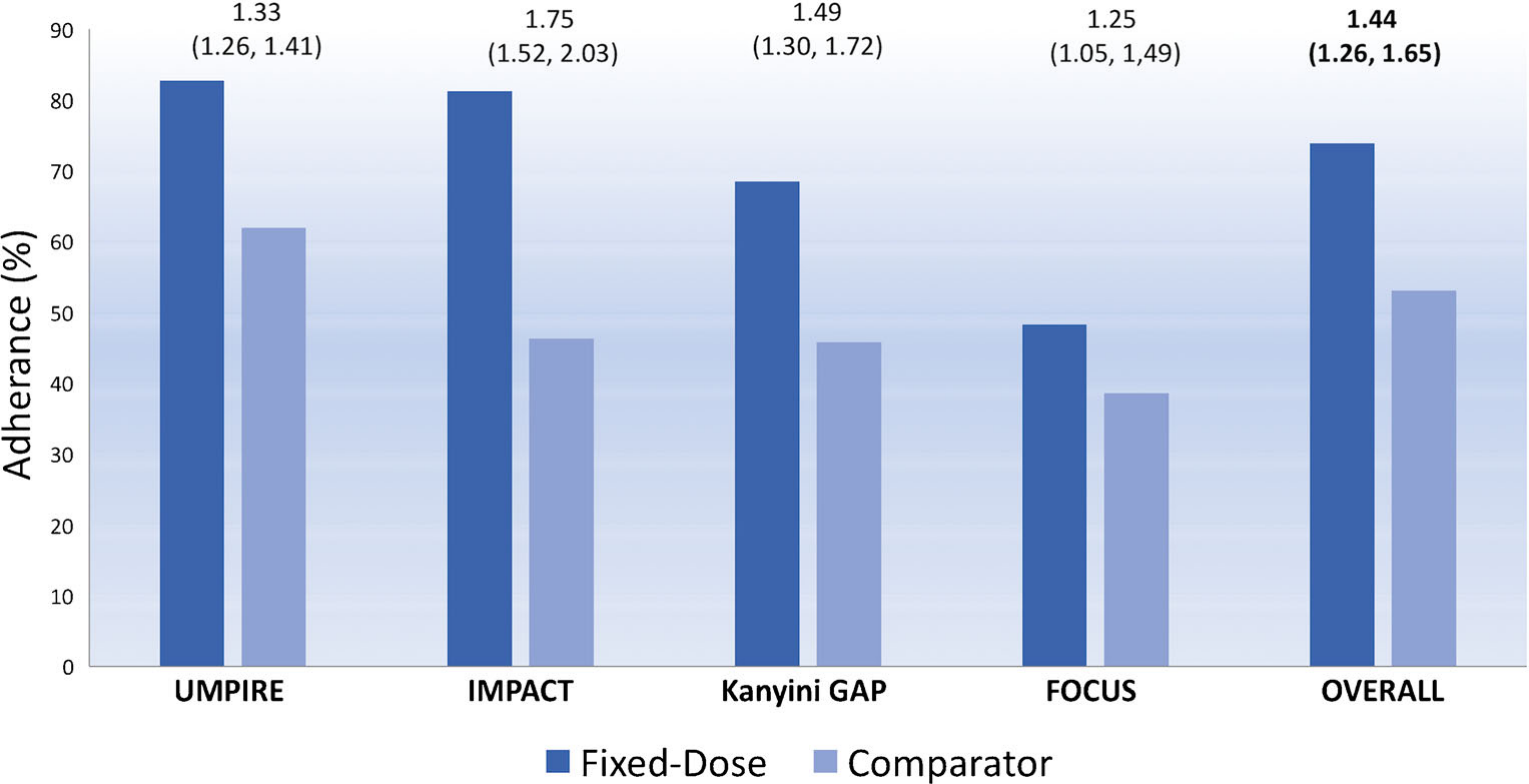
Percentage of adherent patients to the polypill and the comparator group in the various trials and the overall group. The relative risk and the 95% confidence interval of each comparison is mentioned above the *bars* [22•]

One of the formidable challenges in management of CVD is the requirement of adherence to medications over a long duration, sometimes life-long, in individuals who may be asymptomatic and thus the immediate benefits of therapy may not be perceived by them. The polypill is conceptualised to reduce pill burden and thus improve adherence. This was confirmed both in the short-term and intermediate to long-term studies done so far. Two short-term studies of 12 weeks comparing polypill to placebo revealed high adherence rates to polypill which was equivalent to placebo suggesting that adherence, at least in the short term, seems related to the pill count and not the constituents of the polypill [13, 14]. The three intermediate to long term studies of over 12 months: UMPIRE Trial [18], Kanyini GAP [19] and IMPACT [12] clearly demonstrated higher adherence in the polypill arm. A meta-analysis of four studies in patients with CVD or at high-risk for CVD demonstrated 44% higher adherence rates among polypill users as compared to usual care [22•] (Fig. 1). The individual participant data analyses from SPACE suggested that participants with low adherence at baseline had the highest improvement in adherence (17% at baseline to 74%at trial end (relative risk [RR] = 4.46 [95% CI: 3.72 to 5.36]), compared with participants who were already adherent at baseline (86 to 90%; RR = 1.04[95% CI; 1.01 to 1.07] [18]. Interestingly, the individuals without clinical CVD had a better risk ratio of adherence on polypill than usual care (2.12 vs. 1.4, *p* < 0.001) as compared to those with CVD in the SPACE study [20••]. The adherence rates also seem to be better with polypill with time as seen in the FOCUS trial [21]. The adherence rates in the usual care and polypill arm at 1 month being 54.8 and 59%, respectively, with the difference increasing to 55.7 and 65.7%, respectively, at 9 months. Thus from available data, it seems that the polypill improves adherence rates, more so in otherwise poorly compliant groups like those with poor baseline compliance, high risk cases on primary prevention and in subjects on long term therapy.

### Quality of Life

The UMPIRE study showed that EQ-5D visual analog scale score was significantly higher in the polypill group (2.43; 95% CI, 0.87–3.99; *p* = .002) as compared to the usual care group indicating better patient perceived health on polypill [18]. However, the same test was not different in Kanyini-GAP study which could be due to the small sample size of this study [19]. Similarly, the third study with the Red Heart Pill, the IMPACT trial, did not reveal any difference in the QOL between groups but it did reveal that the polypill patients found their medication regime ‘very easy’ (53% of polypill patients compared to 46% of usual therapy patients) [12]. This was also supported in the short-term Red Heart Pill study, where the polypill was preferred over usual care in 92% of patients in the TEMPUS trial [17].

### Cardiovascular Risk Factor Control

Polypill combines multiple drugs within one tablet/capsule. While it is one thing to know the right pharmacological agents for primary and secondary prevention of CVD, it is important to be sure of the physico-chemical compatibilities of these agents within a combination pill. It is very important to conserve the biopharmaceutical and pharmacokinetic properties of every one of its components to have the desired pharmacodynamic effects needed for control of the targeted risk factor. In this context again there have been several studies both short-term phase II studies and clinical trials in real world settings. The meticulously designed TIPS-1 study was a phase II study to assess the effect of five drug polypill (Aspirin 100 mg, simvastatin 20 mg, ramipril 5 mg, hydrochlorthiazide 12.5 mg and atenolol 50 mg) against eight groups comprising of aspirin alone, simvastatin alone, hydrochlorthiazide alone, three combinations of the two blood-pressure-lowering drugs, three blood-pressure-lowering drugs alone or three blood-pressure-lowering drugs plus aspirin [15]. The study revealed that at the end of 12 weeks, the blood pressure effect and the change in11-dehydrothromboxane B2, a surrogate for aspirin effect, were similar in polypill and other groups. The blood pressure lowering effect increased as the number of drugs increased being 2.2/1·3 mm Hg with one drug, 4.7/3·6 mmHg with two drugs and 6.3/4·5 mmHg with three drugs and 7.4/5.5 mmHg with the polypill. The reduction in 11-dehydrothromboxane B2 were similar with the polypill (283·1 ng/mmol creatinine, 95% CI 229·1–337·0) compared with the three blood-pressure-lowering drugs plus aspirin (350·0 ng/mmol creatinine, 294·6–404·0), and aspirin alone (348·8 ng/mmol creatinine, 277·6–419·9). However, the reduction in LDL cholesterol was lower with polypill; 0·70 mmol/L (95% CI 0·62–0·78) vs. 0·83 mmol/L (0·72–0·93; *p* = 0·04) seen with simvastatin alone; with both reductions being greater than for groups without simvastatin (*p* < 0·0001). The drug discontinuity rate of polypill was similar to that of other treatments, with no evidence of increasing intolerance with increasing number of active components in one pill [15].

Another randomised double-blind placebo-controlled crossover trial conducted by Wald et al. revealed higher blood pressure reduction and LDL cholesterol reduction which was closer to the predicted changes suggested in the early papers on polypill. This polypill comprised of amlodipine 2.5 mg, losartan 25 mg, hydrochlorothiazide 25 mg and simvastatin 40 mg and led to a reduction in systolic blood pressure of 17.9 mmHg (95% CI, 15.7–20.1); diastolic blood pressure of 9.8 mmHg (95% CI, 8.1–11.5) and LDL cholesterol of 54.1 mg/dl (95% CI, 46.4 mg/dl −61.9 mg/dl) as compared to placebo. The larger effect was probably due to choice and dose of anti-hypertensives, higher dose of simvastatin and cross-over design of the trial [14].

Early polypill trials in clinical settings comparing polypill to usual care like IMPACT [12], TEMPUS [17] and Kanyini-GAP [19] did not observe any significant difference between the two groups in terms of BP and LDL cholesterol reduction despite reduced pill counts and improvement in adherence. However, most of these were small studies without sufficient statistical power to detect this benefit. The larger UMPIRE Trial demonstrated better blood pressure and cholesterol lowering throughout the study with end of study SBP and LDL-C being 2.6 mmHg and 4.2 mg/dl, respectively, lower in the polypill arm [18]. These findings were corroborated by SPACE meta-analysis which revealed 2.5 mmHg lower SBP and 3.5 mg/dl lower LDL-cholesterol in polypill arm [20••].

Thus, the polypill seems to be working better than usual care for risk factor control. Further refinement in the choice of anti-hypertensives and use of more potent statins in the future iterations would lead to even better results.

## Limitations

### Titration of Doses

Clinicians by training are taught to titrate dosages to ‘treat to target’ various risk factors. The key worry for most practitioners has been the inconvenience of dose adjustments with a polypill. The lack of ability to fine tune one component of the polypill if the therapeutic goals are not reached is a constant concern. However, the more recent guidelines have shifted to risk-based treatment for lipids irrespective of baseline cholesterol levels [23], and others have advocated the same for blood pressure [24]. This approach was tested for intermediate risk individuals without CVD in the HOPE-3 trial. The study included men above 55 years and women above 65 years with one additional cardiovascular risk factor. In a 2 × 2 factorial design individuals received rosuvastatin 10 mg/day or placebo and fixed combination of candesartan (16 mg per day) and hydrochlorothiazide (12.5 mg per day) or placebo. The trial demonstrated that individuals on dual therapy versus those on dual placebo had a 29% reduction (hazard ratio, 0.71; 95% confidence interval [CI], 0.56 to 0.90; *p* = 0.005) in death from cardiovascular causes, non-fatal MI and non-fatal stroke. While the lipid lowering arm had 24% reduction (hazard ratio, 0.76; 95% confidence interval [CI], 0.64 to 0.91; *p* = 0.002) in the combined end point, the benefit in the blood pressure lowering arm was restricted to the top tertile with SBP above 143.5 mmHg [25].

### Impact of Drug Intolerance

The other concern with polypill has been that a side-effect of any one of the components can lead to discontinuation of all drugs. A Cochrane review revealed that discontinuation rates were 26% (95% CI 1.02 to 1.55) higher in participants who were randomised to polypill [26] but comparator groups included participants receiving usual care and placebo. However, the long-term studies showing better risk factor control suggests that this increased discontinuation was offset by other advantages of polypill including better adherence.

### Low Acceptability among Physicians

The buzz over polypill has now been around for several years. The studies with polypill have also been published, and in some countries like India it has been available in store; however, its use has not picked up. Polypills with more than two drugs have not picked up commercially, and the reasons for this need to be explored.

### Mass Medicalisation

The other concern of polypill, since the first paper by Wald and Law on treating everyone above 50 years of age, was the mass medicalisation of the population. This would lead to rapid escalation in numbers qualifying to receive polypill. Another feared fallout of this approach is the poor compliance and non-adherence to life-style advice among polypill users. However, this is not borne out in the available clinical trials. In the UMPIRE Trial, the lifestyle changes were similar in the polypill group and the usual care group and showed improvement from baseline [18].

## Future Directions

### Research

The polypill has been an area of active research over the last decade as was discussed above. However, none of the studies performed so far were powered to detect the impact of polypill on cardiovascular events and mortality. The meta-analysis by SPACE collaborators revealed similar fatal and non-fatal cardiovascular outcomes during follow-up (5.9% in polypill and 4.8% usual care arm, *p* = 0.18). The all cause (1.6 vs. 1.8%, *p* = 0.59) and cardiovascular mortality (1.1 vs. 0.8%, *p* = 0.27) in the polypill and usual care arm, respectively, were also similar [20••]. Ongoing studies with larger patient population and longer follow-up like TIPS-3 [27], SECURE trial [28] and PolyIran trial [29] may be able to address this question and tease out the difference between these two approaches in the future. Importantly, the TIPS-3 trial is a purely primary prevention trial in medium to high risk individuals and thus will help understand the role of polypill in subjects without CVD. The study employs a 2 × 2 × 2 factorial design testing whether polypill compared to placebo leads to prevention of cardiovascular death, stroke and MI in male participants aged over 55 years and female participants over 60 years and INTERHEAT risk score above 10. The study would recruit 5000 subjects from ten different countries and follow up for about 5 years [27]. The SECURE trial on the other hand is a secondary prevention trial and will assess the potential of the polypill to prevent major cardiovascular events in 3206 elderly patients (aged >65 years) with recent MI, stroke or coronary revascularisation over a minimum period of 2 years [28]. The third large trial is the PolyIran trial being conducted exclusively in a developing country to assess the role of polypill in primary and secondary prevention. The study is expected to enrol 7000 participants over 50 years of age and follow them up over 5 years to document time elapsed before the first cardiovascular event [29].

The polypill, especially in primary prevention, also needs to be tested against a multi-component life-style intervention (Poly-lifestyle) like regular physical activity, healthy diet and optimum body weight to see their respective roles in preventing cardiovascular events. While the two approaches may not be mutually exclusive, it may be worthwhile testing the benefits of the two interventions in isolation and combination.

### Customisation of the Polypill

The lack of enthusiasm after the initial hype for the prescription of the polypill even in countries where it is widely available, like India, could be due to lack of customisation of the CVD drugs for individual patients. A common polypill for all CVD prevention subsets is probably viewed as a shotgun approach by practitioners. There may thus be a need for customisation of the polypill into broad categories like those for CHD, stroke and high risk primary prevention. The polypill for secondary prevention among stroke patients has little basis for beta-blocker as one of the constituents. The polypill in this case should have aspirin, statin with diuretic or ACE inhibitors or both depending on the baseline blood pressure level [30]. The polypill for CAD could include aspirin, high dose potent statin, ACE inhibitors and beta-blockers. The polypill should be customised for high risk primary prevention based on baseline blood pressure levels in the light of the HOPE-3 blood pressure lowering trial, which showed benefit of blood pressure reduction only in the individuals in the highest tertile with high blood pressure [25]. Similarly, in these patients polypill with and without aspirin should be available to guide therapy based on individual risk of patients. In summary, a choice of four to six polypills instead of one, for different subsets of patients, may improve uptake of polypill prescription among physicians.

### Incorporation in the Health System

World Health Organisation has outlined nine targets for reducing premature mortality from non-communicable diseases by 25% by 2025. Two of these directly relate to pharmacotherapy in (i) ensuring at least 50% of eligible people receive drug therapy to prevent heart attack and stroke and (ii) 80% availability of the affordable essential medicines, including generics, required to treat major NCDs in both public and private facilities [31]. However, the PURE study revealed that less than 50% of patients with CVD in high income countries and less than 10% of patients in low income countries received the three or more proven effective drugs for secondary prevention [32]. The reasons for this poor rates of therapy was both non-availability and unaffordability of these medicines in upper middle-income, lower middle-income and low-income countries [33]. The polypill is thus well suited to fill this void and provide alternative cardiovascular preventive therapeutic strategy as the current mode of administering secondary prevention does not seem to be working. The affordability and availability of these medicines could be improved by providing them through a publically financed or subsidised universal health coverage programme. Large scale-pooled public procurement of the drug could help substantially drive down the prices.

## Conclusions

In its journey of the past decade, the polypill has travelled from a hyped concept to attaining acceptability in the competitive world of pharmacotherapeutics. The available studies do seem to favour the polypill in terms of improving adherence and reducing the cardiovascular risk burden of high blood pressure and dyslipidemia. The extent of its impact on major cardiovascular events would become evident in the near future through large trials with outcome endpoints. If positive, this would probably increase its acceptability among physicians and health administrators to unreservedly accept it in their armamentarium to fight the mounting burden of CVD. In the meanwhile, strategies to prevent CVD through improved behaviours and judicious use of available drugs must be implemented effectively through an efficient health system. The polypill can fit well in to such a system but cannot substitute for it.
